# Enhanced Bioremediation of Soil Artificially Contaminated with Petroleum Hydrocarbons after Amendment with *Capra aegagrus hircus* (Goat) Manure

**DOI:** 10.1155/2015/657349

**Published:** 2015-12-06

**Authors:** T. P. Nwogu, C. C. Azubuike, C. J. Ogugbue

**Affiliations:** Department of Microbiology, Faculty of Science, University of Port Harcourt, East West Road, PMB 5323, Choba, Port Harcourt 500004, Rivers State, Nigeria

## Abstract

This study was carried out to evaluate the biostimulant potentials of* Capra aegagrus hircus* manure for bioremediation of crude oil contaminated soil (COCS) under tropical conditions. 1 kg of COCS sample was amended with 0.02 kg of* C. a. hircus* manure and monitored at 14-day intervals for total petroleum hydrocarbon (TPH), nutrient content, and changes in microbial counts. At the end of the study period, there was 62.08% decrease in the concentration of TPH in the amended sample compared to 8.15% decrease in the unamended sample, with significant differences (*P* < 0.05) in TPH concentrations for both samples at different time intervals. Similarly, there was a gradual decrease in the concentrations of total organic carbon, nitrogen, phosphorus, and potassium in both samples. The culturable hydrocarbon-utilizing bacteria (CHUB) increased steadily from 8.5 × 10^5^ cfu/g to 2.70 × 10^6^ cfu/g and from 8.0 × 10^5^ cfu/g to 1.78 × 10^6^ cfu/g for both samples.* Acinetobacter*,* Achromobacter*,* Bacillus*,* Flavobacterium*,* Klebsiella*,* Micrococcus*,* Pseudomonas*, and* Staphylococcus *were isolated from amended sample with* Pseudomonas* being the predominant isolated bacterial genus. This study demonstrated that* C. a. hircus* manure is a good biostimulant, which enhanced the activities of indigenous hydrocarbonoclastic bacteria resulting in significant decrease in TPH concentration of COCS.

## 1. Introduction

The contamination or pollution of any environment with crude oil and related products poses a threat to the life of aquatic and terrestrial organisms including humans living in such polluted environments. This is due to the potential toxicity, mutagenicity, and carcinogenicity of crude oil products when present in high concentrations [1]. The potential dangers [2, 3] resulting from crude oil pollution have driven man into the search for environmentally friendly approaches to reclaim polluted environments, particularly crude oil polluted soil. Crude oil is known to contain a complex mixture of natural occurring hydrocarbons (aliphatic, alicyclic, and aromatic), which can be refined into diesel, gasoline, heating oil, jet fuel, kerosene, and other products (petrochemicals) of economic importance [4–7]. These dangers in combination with others can alter population dynamics and disrupt trophic interactions and structure of natural communities within an ecosystem [8].

Bioremediation techniques are noninvasive, cost-effective, and environmentally friendly compared to their counterparts (physical and chemical methods of remediation) [9, 10]. In addition, bioremediation conserves soil texture and characteristics [11–13]. Although bioremediation has many advantages, the success of its application depends on complex factors, which are classified into two major groups, namely, nature of pollutant (degree of pollution, aggregate, and oxidation state of crude oil) and environmental conditions (temperature, oxygen concentration, climatological conditions, pH, moisture content, presence of alternate carbon sources and microbes with degradation capability, soil property, and nutrient availability) [7, 14, 15]. It is paramount that, in any polluted environment, appropriate nutrient concentrations especially nitrogen and phosphorus are maintained in the optimal ratio to offset the imbalance caused by high carbon content of crude oil during pollution, which may retard the growth and activities of hydrocarbonoclastic bacteria [9, 15, 16].

Over the years, synthetic fertilizers have been applied as biostimulants for enhanced bioremediation of petroleum hydrocarbon polluted sites. However, its excessive application has been implicated in negative consequences such as eutrophication, blue baby syndrome, and atmospheric pollution [17]. Moreover, synthetic fertilizers are very expensive in developing countries like Nigeria due to their high demand as an essential agricultural input [18]. These challenges and the quest for environmental sustainability have motivated researchers to search for organic substrates, which would be used as alternatives to synthetic fertilizers to enhance bioremediation. Currently, nitrogen-rich organic substrates are being used as biostimulants, and they have proven to be useful in enhancing the rate of bioremediation. Some of these nitrogen-containing organic substrates, which have been used as biostimulants, include corn residues, sugarcane bagasse, banana skin, yam peel, saw dust, spent brewing grain, rice husk, coconut shell, cow manure, pig manure, and poultry manure [4, 7, 19–24].

In line with this current trend, this study was carried out to evaluate and explore the feasibility of using* Capra aegagrus hircus* (goat) manure, which is widely distributed in Eastern Nigeria, as a nutrient supplement for enhanced bioremediation of crude oil contaminated soil (COCS).

## 2. Materials and Methods

### 2.1. Sample Collection

Pristine loam soil samples were collected at depth of 20 cm from the University of Port Harcourt Botanical Garden, University Park, and they were mixed to obtain composite samples. Following this, samples were transported to laboratory in a precleaned polyethylene bag and were stored at 4°C until analyses were carried out [25]. The crude oil used in this study was obtained from the Department of Petroleum Resources (DPR), Port Harcourt City, while the* C. a. hircus* manure was obtained from Mile III Market, Port Harcourt. All other chemicals, reagents, and media were of analytical grade and were purchased from Sigma-Aldrich, USA.

### 2.2. Determination of the Physicochemical Parameters of Soil

The physicochemical properties of the soil were determined as follows: electrical conductivity [26]; moisture content [27]; total organic carbon, modified wet combustion method [28]; total nitrogen, semimicro Kjeldahl method [29]; available phosphorus, Bray's No. 1 method [30]; and available potassium, atomic absorption spectrophotometer (AAS) following acid digestion. In brief, digestion was carried out by mixing 1 g of each soil sample with 10 mL of acid digestion mixture and heated until there was evolution of white fumes. Following this, samples were allowed to cool to 45°C and after filtration sample volumes were adjusted to 50 mL with distilled water. The digestates were further analyzed using AAS (Biotech-896, UK) and the concentration of potassium was calculated from standards; the resulting values were further multiplied by the dilution factor (50). The pH and temperature were determined using pH meter (WinLab digital pH meter, Germany) and thermometer (MRC 201, Israel), respectively. These procedures were repeated for the amended and control samples at interval of 14 days during the study period. Following the base line analyses as described above, an amended sample was set up by spiking 1 kg of pristine soil sample with 50 mL of crude oil and 20 g of* C. a. hircus* manure [31]. However, in the control sample (unamended), no* C. a. hircus* manure was added.

### 2.3. Analysis of Total Petroleum Hydrocarbon (TPH)

The TPH of the samples were analyzed as previously described [32, 33]. The calibration of the chromatographic column was done using commercially available TPH primary standards (AccuStandard, USA) and the peaks were used to quantify the TPH concentrations in the soil samples. Briefly, Analar grade hexane and acetone (1 : 1, v/v) in an extraction bottle, equipped with Teflon cover, were used as the extraction solution. Each sample was sonicated for 1 h and the two phases were separated by decantation. Extracted organic phases were normalized to 1 mL using vacuum rotary evaporator. Following this, 1 *μ*L of the final extraction solution was injected and eluted in already calibrated gas chromatographic column (HP 5890) as described above.

### 2.4. Enumeration of Bacteria in COCS

The isolation of total culturable heterotrophic bacteria (TCHB) and culturable hydrocarbon-utilizing bacteria (CHUB) was carried out as described [3, 9]. In brief, 1 g of each sample was aseptically transferred into conical flask containing 9 mL of diluent (0.85% NaCl) and was serially diluted. Thereafter, 0.1 mL of each dilution was aseptically transferred onto plate count agar in duplicates and incubated at 37°C for 24 h. However, for CHUB, mineral salt medium (Bushnell-Haas) was used and plates were incubated at 28°C for 7 days using the vapour-phase transfer method. The CHUB were identified based on their morphological features and biochemical characteristics with reference to Bergey's Manual of Determinative Bacteriology [34].

### 2.5. Data and Statistical Analyses

The TPH concentrations were calculated as previously described [7]. One-way analysis of variance (ANOVA) was used to determine the level of significance of decreasing TPH concentrations in the amended and unamended samples.

## 3. Results

### 3.1. Physicochemical Properties of Amended and Unamended Samples

Samples were analyzed for several physicochemical parameters at 14-day intervals during the study period. The baseline data showed that the pH of the* C. a. hircus* manure, pristine soil, amended samples, and unamended samples were all slightly acidic (Table 1). However, the pH of the amended and unamended samples increased slightly during the first 28 days of study before a subsequent gradual decrease was obtained towards the end of the study period. The ambient temperature was 28.0 ± 0.5°C throughout the study period. In the amended sample, the total organic carbon decreased by 16.41% as compared to 4.6% decrease obtained in the unamended sample. Similarly, the concentrations of total nitrogen, available phosphorus, and available potassium decreased by 11.67%, 13.84%, and 19.02%, respectively, for amended samples and by 7.02%, 13.58%, and 13.03%, respectively, for the unamended sample (Tables 2 and 3).

### 3.2. Analysis of Total Petroleum Hydrocarbon

**Figure 1:**
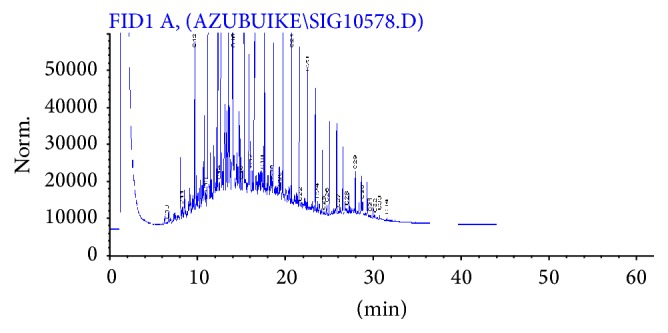
Chromatogram of crude oil sample used in this study. The peaks represent residual carbon fractions detected in the sample prior to the study.

**Figure 2:**
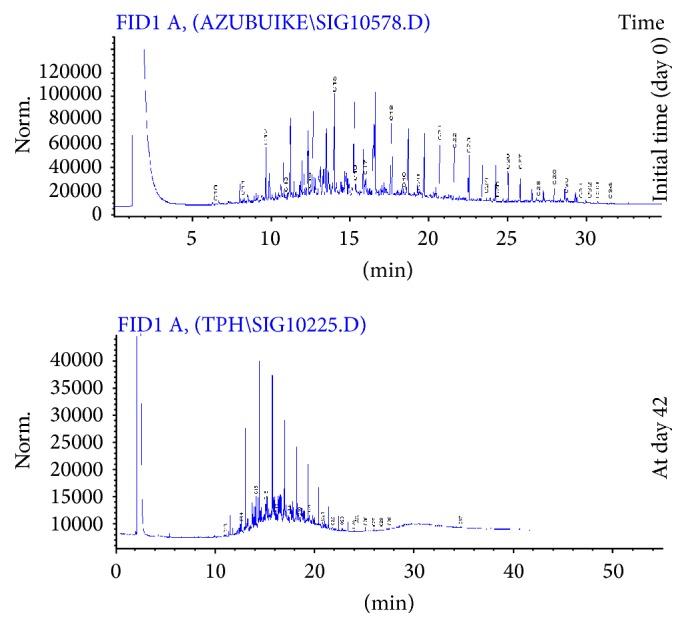
Gas chromatograms of amended samples obtained at day 0 and day 42 during the study period. The peaks represent residual carbon fractions in the samples during the study.

**Figure 3:**
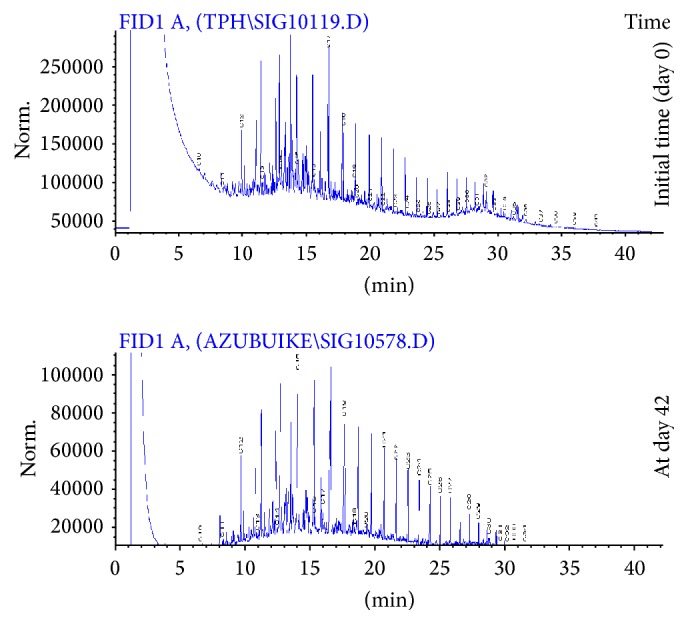
Gas chromatograms of unamended samples obtained at day 0 and day 42 during the study period. The peaks represent residual carbon fractions in the samples during the study.

**Figure 4:**
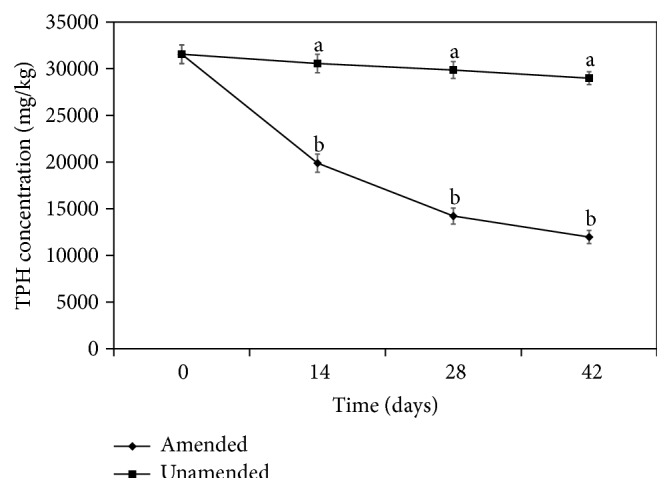
Changes in TPH concentrations of amended and unamended samples obtained during the study period. Bars represent standard errors, *n* = 2; different letters represent significant differences (*P* < 0.05).

The application of gas chromatography allowed detection of carbon fractions (C_10_–C_34_) in the crude oil sample (Figure 1). The chromatogram obtained at day 42 of the study shows that more than half of the carbon fractions of the crude oil in the amended sample were attenuated (Figure 2). However, at day 14 and day 28, the peaks indicating the various fractions were almost identical (Figure not shown) but with different TPH concentrations which indicated 28.52% decrease in TPH concentration within this particular time interval. Similarly, for the unamended sample, reduction in TPH fractions was gradual at day 14, day 28, and day 42, albeit little or no changes were observed in the magnitude of the peaks obtained in the gas chromatograms during the stated period. The decreased percentages in TPH concentration in the unamended COCS between day 14 and day 28 and between day 28 and day 42 were 2.26% and 2.95%, respectively (Figure 3). There were significant differences (*P* < 0.05) in TPH concentrations obtained at different time intervals in the amended sample (Figure 4). Generally, the decrease in TPH concentration in amended and unamended samples were 62.08 and 8.15%, respectively, by the end of the study.

### 3.3. Microbiological Analyses

**Figure 5:**
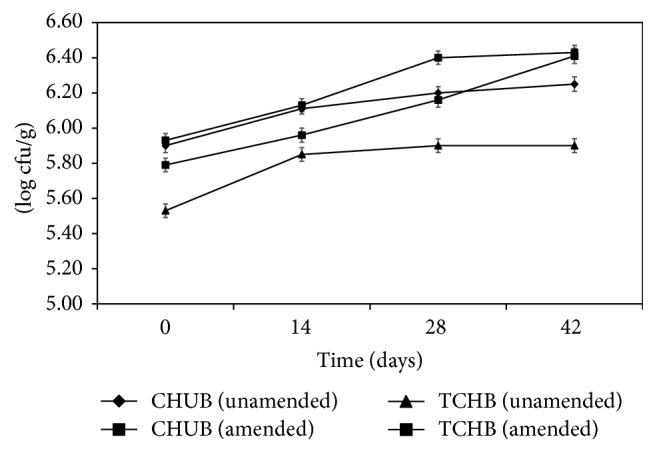
Changes in microbial counts (log cfu/g) of amended and unamended samples during the study period. Data indicate the rapid proliferation of culturable hydrocarbon-utilizing bacteria (CHUB) and total culturable heterotrophic bacteria (TCHB) in the amended sample. Bars represent standard errors, *n* = 2.

The TCHB of amended and unamended samples increased steadily from 6.2 × 10^5^ to 2.6 × 10^6^ cfu/g and from 3.4 × 10^5^ to 8.0 × 10^5^ cfu/g, respectively, during the study period. Likewise, the CHUB increased from 8.5 × 10^5^ to 2.70 × 10^6^ cfu/g and from 8.0 × 10^5^ to 1.78 × 10^6^ cfu/g for amended and unamended samples, respectively (Figure 5). The bacterial isolates obtained were identified based on their colonial morphology, microscopic morphology, and biochemical characteristics with reference to Bergey's Manual of Determinative Bacteriology. The identified genera were* Acinetobacter*,* Achromobacter*,* Bacillus*,* Flavobacterium*,* Klebsiella*,* Micrococcus*,* Pseudomonas*, and* Staphylococcus.* Amongst the isolates,* Micrococcus* and* Pseudomonas* were the predominant bacterial isolates, while* Achromobacter* and* Flavobacterium* were less frequently isolated.

## 4. Discussion

Crude oil contaminated soil sample was amended with* C. a. hircus* manure and monitored for decreasing total petroleum hydrocarbon (TPH) concentration for a period of 42 days. The significant decrease in TPH concentration in the amended sample compared to the unamended sample at different time intervals was as a result of the additional nutrient (N, P, and K) contained in the* C. a. hircus* manure. These nutrients are the basic building blocks of life, which stimulated microbial growth and allowed microbes to synthesize the necessary enzymes needed to break down the petroleum hydrocarbon contaminants [11]. Although microorganisms are present in contaminated soil, their numbers might not be sufficient to initiate remediation of contaminated sites. The growth and activities of hydrocarbonoclastic bacteria must be stimulated and all require nitrogen, phosphorus, and carbon as building blocks. Previous studies have demonstrated that nitrogen is essential for cellular protein and cell wall configuration, while phosphorus is needed for nucleic acids, cell membrane, and ATP formation [35]. Therefore, bioremediation of contaminated crude oil sample requires an adequate supply of these elements, which in turn are utilized by crude oil degrading microorganisms for their active growth and metabolic performance [36].

Nutrient limitation in hydrocarbon-contaminated soils presents a challenge to bioremediation; nonetheless, addition of nutrients generally benefits soil hydrocarbonoclastic bacteria resulting in enhanced bioremediation of hydrocarbon-polluted environment [37, 38]. The nutritional requirements of hydrocarbonoclastic bacteria in terms of carbon to nitrogen ratio and carbon to phosphorus ratio were observed to be 10 : 1 and 30 : 1, respectively [11]. Studies [39] have also reported that soil amendment with C : N ratio of 10 : 1 significantly increased polyaromatic hydrocarbon (PAH) biodegradation compared to those with C : N ratio ≥ 25 : 1. In this present study, the C : N : P ratio in the amended sample was 10 : 2 : 1 throughout the study period, while in the unamended sample the C : N : P ratios were 54 : 1 : 1 (day 0), 58 : 1 : 1 (day 14), and 60 : 1 : 1 (day 28 and day 42). The carbon ratio in the unamended sample was obviously higher than that in the amended sample, and this imbalance neither favoured nor stifled the activities of the hydrocarbon-utilizing bacteria (HUB) in the unamended sample. This observation is in agreement with Liebig's law of the minimum, which states that growth is controlled not by the total amount of resource available but by the scarcest resources (limiting factor) [40], which are, in this case, N and P. These nutrients (N and P) in the appropriate ratio favoured the proliferation of microorganisms in the amended sample and the activities of these microbes resulted in the decrease in TPH concentration obtained [4, 31, 41]. Similarly, previous studies demonstrated that degradation of crude oil was also enhanced when other animal manures were used as biostimulants [42, 43].

The gas chromatogram (GC) of the amended sample showed ≥62% reduction in TPH concentration and a progressive attenuation in peaks of fractions from day 0 to day 42, which suggested that the goat manure was effective as a biostimulant for enhanced bioremediation of crude oil contaminated soil (COCS). In the unamended sample, many of the TPH fractions were not attenuated, even at day 42. Hence, the rapid attenuation of the fractions in amended sample (Figure 2) was a clear indication of petroleum hydrocarbon degradation by the soil microbial community. The attenuation of the peaks of the different carbon fractions in the chromatograms suggested that the petroleum hydrocarbons were mineralized ultimately into carbon dioxide, which was released into the atmosphere. Hydrocarbon utilizing microorganisms mineralize TPH into harmless carbon dioxide via rubredoxin-NADH oxidoreductase reactions during catabolic reactions for energy generation. No new peaks were observed, which would have suggested that the TPH were transformed into simpler substances that might be less or more toxic. Carbon fractions in the range of C_2_–C_10_ chain length could not be detected by the GC analysis at day 0, and this was expected as carbon fractions of chain lengths less than C_8_ are vaporous and could have volatilized from the soil sample soon after contamination.

In this study, the total culturable heterotrophic bacteria (TCHB) in amended and unamended samples increased in population with time. Nevertheless, the counts were higher in amended samples compared to unamended samples at respective intervals. An increase in the population of culturable hydrocarbon-utilizing bacteria (CHUB) was also observed during the study period regardless of the slight proliferation stall observed between day 28 and day 42. A similar study attributed the proliferation to additional microbial populations, which utilized intermediate products from hydrocarbon breakdown [2, 44]. The stall in proliferation of CHUB observed toward the end of the study period was due to depletion in limiting nutrient (N : P), which in turn reduced microbial activities (growth) in the amended sample towards the end of the study [1, 45]. In spite of this stall, a considerable decrease (16%) in TPH concentration was observed between day 28 and day 42. This decrease in TPH concentration without a substantial upward microbial shift is not unusual. A previous study [46] had reported that rapid changes in TPH of contaminated soils might not always be accompanied by changes in microbial community. In another study, [31] reported that CHUB population tended to be highest during the mid-period and decreased gradually towards the end of study. Nevertheless, the observed decrease in microbial count was attributed to decreasing bioavailability of hydrocarbons to the indigenous microflora.

The microbial counts recorded in this study were in the range of 3.4 × 10^5^ cfu/g to 2.70 × 10^6^ cfu/g, which has also been previously reported [47]. Some of the bacterial genera isolated,* Acinetobacter*,* Bacillus*,* Pseudomonas*, and* Micrococcus*, have been reported by other researchers as hydrocarbon utilizing bacteria isolated during bioremediation of crude oil contaminated soils [4, 31, 36, 48]. Although there was no amendment in the control treatment, there was a gradual decrease in the TPH concentration which was relatively slow compared to the events in the amended sample. This observed decrease in TPH concentration (8%) might have largely been due to other processes such as volatilization, adsorption, and abiotic factors (temperature and pH), which have been reported to contribute to decreasing TPH concentration [31]. Studies have also reported that a slight increase in pH from acidic to alkaline range, in response to organic waste (biostimulant) addition, increased degradation of crude oil in contaminated sites [31, 49]. This is in agreement with the result of this present study where there was an increase (2.13%) in pH from day 0 to day 28. Within this time frame, relatively higher reductions in TPH concentrations were obtained as follows: 36.99% (day 0 to day 14) and 28.52% (day 14 to day 28). However, between day 28 and day 42 when the pH decreased slightly, the percentage of decrease in TPH was only 15.80%.

## 5. Conclusion

This study demonstrated that* C. a. hircus* manure is a good organic substrate containing nitrogen, phosphorus, and potassium, which have great potentials for enhanced bioremediation of COCS.* Capra aegagrus hircus* manure when applied in appropriate concentration to COCS would enhance the bioremediation of such sites by increasing microbial activities of autochthonous microflora following the slow release of nutrients. Interestingly, the decrease in TPH and peak attenuations obtained were just in 42 days of monitoring, which suggests that the use of* C. a. hircus* manure could be a cost-efficient and process-efficient bioremediation option for hydrocarbon-contaminated soils. The availability of* C. a. hircus* manure in commercial quantity in Nigeria makes this waste product a potential and viable biostimulant for enhanced bioremediation of crude oil polluted environments.

## Figures and Tables

**Table 1:** Physicochemical properties of pristine soil sample and *C. a. hircus* manure.

Parameters	Pristine soil	*C. a. hircus* manure
pH	6.63	6.20
Temperature, °C	28.1	27.8
Electrical conductivity, *µ*S/cm	256.3	6990
Moisture content, %	11.1	—
Total organic carbon, mg/kg	219.20	10420
Total nitrogen, mg/kg	ND	3510
Available phosphorus, mg/kg	ND	2554
Available potassium, mg/kg	ND	1398

**Table 2:** Changes in physicochemical properties of amended soil sample.

Parameters	Day 0	Day 14	Day 28	Day 42
pH	6.11	6.19	6.24	6.20
Temperature, °C	28.0	28.5	28.2	28.0
Electrical conductivity, *µ*S/cm	1405	1325	1304	1287
Total organic carbon, mg/kg	4558	3955	3868	3810
Total nitrogen, mg/kg	850.45	785.31	763.22	751.26
Available phosphorus, mg/kg	435.44	401.28	385.04	375.17
Available potassium, mg/kg	303.98	277.88	259.63	246.17
TPH, mg/kg	31550	19880	14210	11965

**Table 3:** Changes in physicochemical properties of unamended soil sample.

Parameters	Day 0	Day 14	Day 28	Day 42
pH	6.64	6.60	6.57	6.59
Temperature, °C	28.1	28.4	28.1	28.3
Electrical conductivity, *µ*S/cm	292.92	288.56	281.44	278.56
Total organic carbon, mg/kg	4200	4159	4088	4004
Total nitrogen, mg/kg	101.22	99.87	96.78	94.11
Available phosphorus, mg/kg	77.59	71.09	68.22	67.05
Available potassium, mg/kg	67.48	62.69	60.44	58.69
TPH, mg/kg	31550	30550	29860	28980

## References

[1] Adebusoye S. A., Ilori M. O., Amund O. O., Teniola O. D., Olatope S. O. (2007). Microbial degradation of petroleum hydrocarbons in a polluted tropical stream. *World Journal of Microbiology and Biotechnology*.

[2] Okpokwasili G. C., Nnorom E. E., Akpata T. V. T., Okali D. U. U. (1990). Microbial degradation of petroleum hydrocarbons by brackish water isolates. *Nigeria Wetlands*.

[3] Chikere C. B., Azubuike C. C. (2013). Catechol-2,3-dioxygenase screening in putative hydrocarbon utilizing bacteria. *International Research Journal of Microbiology*.

[4] Agarry S. A., Owabor C. N., Yusuf R. O. (2010). Bioremediation of soil artificially contaminated with petroleum hydrocarbon mixtures: evaluation of the use of animal manure and chemical fertilizer. *Bioremediation Journal*.

[5] Agarry S. E., Owabor C. N., Yusuf R. O. (2012). Enhanced bioremediation of soil artificially contaminated with kerosene: optimization of biostimulation agents through statistical experimental design. *Journal of Petroleum and Environmental Biotechnology*.

[6] Iyagba A. G., Offor U. S. (2014). Effect of crude oil and biostimulant (bioremediation) on growth extract of maize (*Zea mays* (l.) and cowpea (*Vignaunguiculata* (l.) Walp). *European Scientific Journal*.

[7] Agarry S. E., Jimoda L. A. (2013). Application of carbon-nitrogen supplementation from plant and animal sources in in-situ soil bioremediation of diesel oil: experimental analysis and kinetic modelling. *Journal of Environment and Earth Science*.

[8] Bejarano A. C., Michel J. (2010). Large-scale risk assessment of polycyclic aromatic hydrocarbons in shoreline sediments from Saudi Arabia: environmental legacy after twelve years of the Gulf war oil spill. *Environmental Pollution*.

[9] Silva-Castro G. A., Uad I., Rodríguez-Calvo A., González-López J., Calvo C. (2015). Response of autochthonous microbiota of diesel polluted soils to land-farming treatments. *Environmental Research*.

[10] Dados A., Omirou M., Demetriou K., Papastephanou C., Ioannides I. M. (2015). Rapid remediation of soil heavily contaminated with hydrocarbons: a comparison of different approaches. *Annals of Microbiology*.

[11] Vidali M. (2001). Bioremediation. An overview. *Pure and Applied Chemistry*.

[12] Adams R. H., Guzmán-Osorio F. J. (2008). Evaluation of land farming and chemicobiological stabilization for treatment of heavily contaminated sediments in a tropical environment. *International Journal of Environmental Science and Technology*.

[13] Yerushalmi L., Rocheleau S., Cimpoia R. (2003). Enhanced biodegradation of petroleum hydrocarbons in contaminated soil. *Bioremediation Journal*.

[14] Gavrilescu M. (2006). Overview of in situ remediation technologies for sites and groundwater. *Environmental Engineering and Management Journal*.

[15] Bamforth S. M., Singleton I. (2005). Bioremediation of polycyclic aromatic hydrocarbons: cruurnt knowledge and future directions. *Journal of Chemical Technology and Biotechnology*.

[16] Ayotamuno M. J., Kogbara R. B., Ogaji S. O. T., Probert S. D. (2006). Bioremediation of a crude-oil polluted agricultural-soil at Port Harcourt, Nigeria. *Applied Energy*.

[17] Geddes B. A., Ryu M.-H., Mus F. (2015). Use of plant colonizing bacteria as chassis for transfer of N_2_-fixation to cereals. *Current Opinion in Biotechnology*.

[18] Danjuma B. Y., Abdulsalam S., Sulaiman A. D. I. (2012). Kinetic investigation of Escravos crude oil contaminated soil using natural stimulants of plant source. *International Journal of Emerging Trends in Engineering and Development*.

[19] Molina-Barahona L., Rodríguez-Vázquez R., Hernández-Velasco M. (2004). Diesel removal from contaminated soils by biostimulation and supplementation with crop residues. *Applied Soil Ecology*.

[20] Okieimen C. O., Okieimen F. E. (2005). Bioremediation of crude oil-polluted soil–effect of poultry droppings and natural rubber processing sludge application on biodegradation of petroleum hydrocarbons. *Environmental Sciences*.

[21] Pala D. M., de Carvalho D. D., Pinto J. C., Sant'Anna G. L. (2006). A suitable model to describe bioremediation of a petroleum-contaminated soil. *International Biodeterioration and Biodegradation*.

[22] Yakubu M. B. (2007). Biodegradation of Lagoma crude oil using pig dung. *African Journal of Biotechnology*.

[23] Akinde S. B., Obire O. (2008). Aerobic heterotrophic bacteria and petroleum-utilizing bacteria from cow dung and poultry manure. *World Journal of Microbiology and Biotechnology*.

[24] Abioye P. O., Abdul Aziz A., Agamuthu P. (2010). Enhanced biodegradation of used engine oil in soil amended with organic wastes. *Water, Air, and Soil Pollution*.

[25] Sojinu O. S. S., Wang J.-Z., Sonibare O. O., Zeng E. Y. (2010). Polycyclic aromatic hydrocarbons in sediments and soils from oil exploration areas of the Niger Delta, Nigeria. *Journal of Hazardous Materials*.

[26] Jackson M. L. (1964). *Soil Chemical Analysis*.

[27] Smith G. N., Smith I. G. N. (1998). *Elements of Soil Mechanics*.

[28] Nelson D. W., Sommer L. E., Page A. L., Miller R. H., Keeney D. R. (1982). Determination of organic carbon. *Methods of Soil Analysis*.

[29] Bremner J. M., Mulvaney C. S., Page A. L., Miller R. H., Keeney D. R. (1982). Total nitrogen determination. *Methods of Soil Analysis*.

[30] Olsen S. R., Sommers L. E., Page A. L., Miller R. H., Keeney D. R. (1982). Determination of available phosphorus. *Methods of Soil Analysis*.

[31] Onuoha S. C. (2013). Stimulated biodegradation of spent lubricating motor oil in soil amended with animal droppings. *Journal of Natural Sciences Research*.

[32] Lladó S., Solanas A. M., de Lapuente J., Borràs M., Viñas M. (2012). A diversified approach to evaluate biostimulation and bioaugmentation strategies for heavy-oil-contaminated soil. *Science of the Total Environment*.

[33] Silva-Castro G. A., Rodelas B., Perucha C., Laguna J., González-López J., Calvo C. (2013). Bioremediation of diesel-polluted soil using biostimulation as post-treatment after oxidation with fenton-like reagents: assays in a pilot plant. *Science of the Total Environment*.

[34] Holt J. G., Krieg N. R., Sneath H. A., Staley J. J., Williams S. T. (1994). *Bergey's Manual of Determinative Bacteriology*.

[35] Swindell C. M., Aelion C. M., Pfaender F. K. (1988). Influence of minerals and organic nutrients anaerobic biodegradation and the adaptation response of surface microbial communities. *Applied Environmental Microbiology*.

[36] Van Hamme J. D., Singh A., Ward O. P. (2003). Recent advances in petroleum microbiology. *Microbiology and Molecular Biology Reviews*.

[37] Beolchini F., Rocchetti L., Regoli F., Dell'Anno A. (2010). Bioremediation of marine sediments contaminated by hydrocarbons: experimental analysis and kinetic modelling. *Journal of Hazardous Materials*.

[38] Kauppi S., Sinkkonen A., Romantschuk M. (2011). Enhancing bioremediation of diesel-fuel-contaminated soil in a boreal climate: comparison of biostimulation and bioaugmentation. *International Biodeterioration and Biodegradation*.

[39] Teng Y., Luo Y., Ping L., Zou D., Li Z., Christie P. (2010). Effects of soil amendment with different carbon sources and other factors on the bioremediation of an aged PAH-contaminated soil. *Biodegradation*.

[40] Gorban A. N., Pokidysheva L. I., Smirnova E. V., Tyukina T. A. (2011). Law of the minimum paradoxes. *Bulletin of Mathematical Biology*.

[41] Adesodun J. K., Mbagwu J. S. C. (2008). Biodegradation of waste-lubricating petroleum oil in a tropical alfisol as mediated by animal droppings. *Bioresource Technology*.

[42] Ijah U. J. J., Antai S. P. (2003). The potential use of chicken-drop micro-organisms for oil spill remediation. *Environmentalist*.

[43] Okolo J. C., Amadi E. N., Odu C. T. I. (2005). Effects of soil treatments containing poultry manure on crude oil degradation in a sandy loam soil. *Applied Ecology and Environmental Research*.

[44] Atlas R. M., Bartha R. (1992). *Microbial Ecology: Fundamentals and Applications*.

[45] Shabir G. M., Afzal M., Anwar F., Tahseen R., Khalid Z. M. (2008). Biodegradation of kerosene in soil by a mixed bacterial culture under different nutrient conditions. *International Biodeterioration and Biodegradation*.

[46] Makadia T. H., Adetutu E. M., Simons K. L., Jardine D., Sheppard P. J., Ball A. S. (2011). Re-use of remediated soils for the bioremediation of waste oil sludge. *Journal of Environmental Management*.

[47] Ijah U. J. J., Antai S. P. (2003). Removal of Nigerian light crude oil in soil over a 12-month period. *International Biodeterioration and Biodegradation*.

[48] Bento F. M., Camargo F. A. O., Okeke B. C., Frankenberger W. T. (2005). Comparative bioremediation of soils contaminated with diesel oil by natural attenuation, biostimulation and bioaugmentation. *Bioresource Technology*.

[49] Ijah U. J. J., Ndana M. (2003). Stimulated biodegradation of crude oil in soil amended with periwinkle shells. *The Environmentalist*.

